# Hand hygiene and facemask use to prevent droplet-transmitted viral diseases during air travel: a systematic literature review

**DOI:** 10.1186/s12889-021-10814-9

**Published:** 2021-04-20

**Authors:** Giulia De Angelis, Franziska Michaela Lohmeyer, Adriano Grossi, Brunella Posteraro, Maurizio Sanguinetti

**Affiliations:** 1 Dipartimento di Scienze Biotecnologiche di Base, Cliniche Intensivologiche e Perioperatorie, Università Cattolica del Sacro Cuore, Rome, Italy; 2 Dipartimento di Scienze di Laboratorio e Infettivologiche, Fondazione Policlinico Universitario A. Gemelli IRCCS, Largo A. Gemelli 8, 00168 Rome, Italy; 3 Direzione Scientifica, Fondazione Policlinico Universitario A. Gemelli IRCCS, Rome, Italy; 4 Dipartimento di Scienze della Vita e Sanità Pubblica, Università Cattolica del Sacro Cuore, Rome, Italy; 5 Dipartimento di Scienze Mediche e Chirurgiche, Fondazione Policlinico Universitario A. Gemelli IRCCS, Rome, Italy

**Keywords:** Air travel, Hand hygiene, Facemask use, Transmission, Viral disease

## Abstract

**Background:**

Transmission of viral diseases (e.g., influenza A H1N1) via respiratory droplets takes place mainly in confined spaces, including in aircraft during commercial air travel. The adoption of hygiene measures may help to prevent disease spread aboard aircraft. This review summarizes the evidence on hand hygiene and the use of facemasks as viral disease prevention measures in aircraft.

**Methods:**

A literature search was performed in the PubMed, Scopus, and Web of Science databases up to 10 June 2020, according to the Preferred Reporting Items for Systematic Reviews and Meta-Analyses criteria. A population, intervention, comparison, outcomes, and study design (PICOS) approach was used to define the review question.

**Results:**

We included four studies published between 2007 and 2020, all targeting influenza virus disease, in the qualitative synthesis. Three studies used mathematical models to simulate single- or multiple-direction flights, and two of them showed that facemask (e.g., N95 respirator) use considerably reduced infection probability. In the third study, hand cleaning by 20 to 60% of people at any time in all airports (including on aircraft) reduced the measure of airports’ power to spread the disease across the globe by ~ 24 to 69%. The fourth study was a case-control study designed to trace an influenza outbreak in two flights during the 2009 influenza A H1N1 pandemic. The study showed that none (0%) of nine infected passengers compared to 15 (47%) of 32 healthy control passengers in the aircraft cabin during one of these flights wore a facemask (odds ratio, 0.0; 95% confidence interval, 0.0–0.7). In contrast, both case and control passengers appeared to be equally compliant in self-assessed hand hygiene.

**Conclusions:**

Facemask use combined with hand hygiene may minimize the chance of droplet-transmitted virus spread by air travelers. Thus, it is necessary that hygiene measures become an integral part of standard procedures in commercial air travel.

**Supplementary Information:**

The online version contains supplementary material available at 10.1186/s12889-021-10814-9.

## Background

In humans, viral respiratory tract infections causing worldwide outbreaks spread largely via respiratory droplets, aerosols, and direct as well as indirect contact transmission. Droplet/aerosol transmission can occur when an infected person ejects large droplets by sneezing, talking, or coughing, which may convert to aerosol particles [1]. Because of their small aerodynamic diameter, these particles come in close contact with a healthy person and are capable of inoculating entry gateways, such as the eye, nose, or mouth. Additionally, particles can deposit on fomites in the direct environment of an infected person, leading to indirect contact transmission, whereas direct contact transmission takes place when the virus passes directly from an infected to a healthy person [2, 3].

Preventive measures of transmission in healthcare settings are addressed in detail in several national and international guidelines, such as the Healthcare Infection Control Practices Advisory Committee (HICPAC) and World Health Organization (WHO) guidelines. In particular, measures include hand hygiene and facemask use to protect patients as well as additional personal equipment, such as medical gloves, gowns, and eye or face shields, to protect personnel in close contact with patients [4, 5].

In contrast, guidelines mainly created in response to an outbreak such as due to influenza A H1N1 in 2009 [6] or severe acute respiratory syndrome coronavirus 2 (SARS-CoV-2)—the etiological agent of coronavirus disease 2019 (COVID-19) [7]—recommend specific nonpharmaceutical prevention measures for individuals and communities in public spaces, also called community mitigation measures [8–10]. Individual precautions include personal protective measures, e.g., hand, respiratory and environmental hygiene, with the aim of reducing individual infection risk. Measures at the community level, such as social distancing or travel-related measures—including travel restrictions or passenger temperature screening—require the involvement of local, regional, or national authorities with the aim of controlling or decelerating international virus transmission. However, these measures can have significant economic, legal, and ethical implications.

Respiratory virus transmission takes place mainly in confined spaces; in addition to healthcare settings (e.g., influenza A H1N1) [11], public transport, including busses and ships [12, 13] and specifically aircraft [14–16], poses a major risk of possible infection. Moser et al. [14] described in 1979 a major influenza outbreak in a 56-seat aircraft, which was blocked for several hours on the ground without fresh air circulation, resulting in the infection of 39 (72.2%) of 54 passengers. Olsen et al. [15] reported the results of three flights with passengers infected by the severe acute respiratory syndrome (SARS) virus; in one (3-h) flight, infections involved 22 (18.3%) of 120 passengers, whereas in the other two (90-min) flights, infections involved 0 (0.0%) of 315 passengers and 1 (0.4%) of 246 passengers. Thus, the authors recommended preventive measures considering travel length and seating distance as relevant factors for possible viral infection [15]. On-line available reports show that the number of flights carried out worldwide reached 38.9 million in 2019 (i.e., in the pre-COVID-19 era) but dropped to 16.4 million in 2020 (i.e., in the COVID-19 era). It is, however, reassuring that despite over 1.2 billion passengers traveling since the beginning of 2020, only 44 cases of COVID-19 reported by the International Air Transport Association (IATA) as of 8 October 2020 have been associated with a flight journey [16]. As discussed below, cabin ventilation and air filtration together—perhaps this was not the case in Moser et al.’s study [14]—ensure that the air aboard modern aircraft is very safe [17]. Likewise, wearing facemasks—which theoretically amplify the air quality in cabins—is necessarily interrupted by meal (or beverage) intake during a flight. As this practice potentially amplifies the onboard risk of viral infection (e.g., related to improper facemask disposal or hand washing performance), it is likely that not serving meals due to 90-min flight duration might have caused negligible infection rates during the two flights in Olsen et al.’s study [15].

Screening of air travelers at entry points from designated/recognized areas of a severe respiratory syndrome by observation, questionnaires, and body temperature assessment in combination with personal protective measures are the most recommended mitigation measures [8, 10, 17]. However, most recommendations have unknown efficacy because of scarce or lacking scientific evidence and rely on studies developed in settings other than aircraft. Specifically, the use of facemasks is controversial.

The present systematic review of the literature summarizes the evidence of hand hygiene and the use of facemasks to prevent droplet-transmitted viral diseases, specifically during travel with aircraft. Furthermore, recommendations and guidelines regarding the applicability of viral disease prevention measures are discussed.

## Methods

In this systematic review of the literature, we followed the Preferred Reporting Items for Systematic Reviews and Meta-Analyses (PRISMA) guidelines [18]. A population, intervention, comparison, outcomes, and study design (PICOS) approach was adopted to define the review question. Studies that involved commercial travel aboard aircraft and that evaluated the impact of hand hygiene and/or facemask use in preventing droplet-transmitted viral diseases in air travelers were considered eligible. No language restrictions were applied, and all study designs were considered eligible. Reviews, commentaries, editorials, and letters were excluded. We searched the PubMed, Scopus, and Web of Science databases for peer-reviewed articles and the gray literature for relevant documents up to 10 June 2020 (the detailed search strategy is reported in the Additional file 1). Additional articles were identified by manually searching the reference lists of the included studies and of reviews dealing with the topic.

**Fig. 1:**
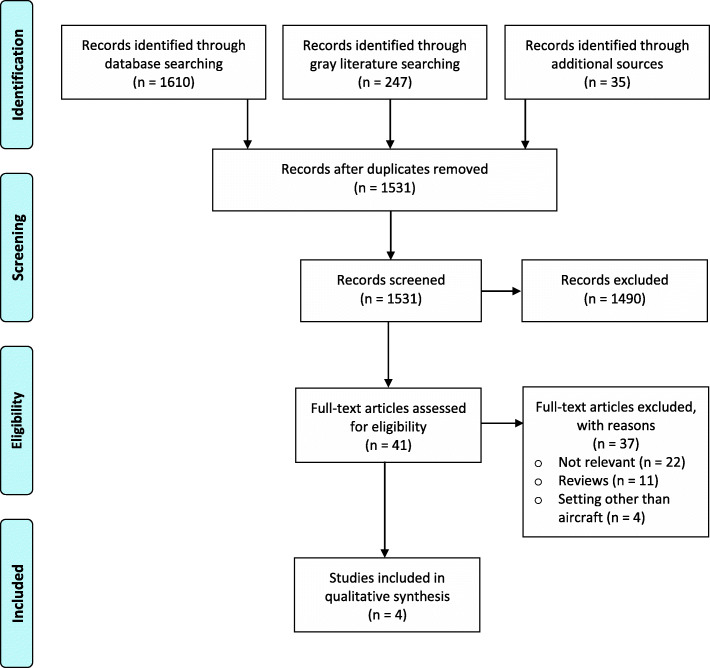
Diagram depicting the PRISMA-based search strategy and the selection process of studies included in the systematic review

After retrieving all studies and removing duplicates, two authors (GDA, FML) independently examined titles and abstracts, and in a second step, full-text articles of studies not meeting the inclusion criteria were discarded (Fig. 1). From each included study, the following data were independently extracted by the two authors and then checked for agreement: first author’s name and year of publication, type of study, type of viral infection, flight details (i.e., number of passengers and duration of flight), hand hygiene strategy, type of facemask, presence of preventive measures other than hand hygiene and facemask wearing, outcome of the study, and main results. In case of disagreement between the two authors, a senior author was consulted (BP).

We evaluated the quality of evidence for air travel-related virus transmission using a bias assessment tool proposed by Leitmeyer et al. [19]. The tool includes the following criteria: i) definitions of index (the first case of infection identified at the beginning of an outbreak) or secondary (susceptible individuals who develop infection after exposure to the index case) cases; ii) strategy, timeliness, and follow-up of contact tracing; and iii) alternative exposure means for infection other than the flight. Based on a score assignment, each study was categorized as having a low (0–3 points), medium (4–6 points), or high (7–9 points) level of evidence.

## Results

The search strategy yielded 1610 records from scientific databases (296 from PubMed, 756 from Scopus, and 558 from Web of Science) and 247 from gray literature sources. An additional 35 articles were manually retrieved from the references of the included studies and of relevant reviews. A total of 1531 titles and abstracts were evaluated after deduplication, with 1490 of them discarded because they were not relevant to the review question. Of the 41 full-text remaining articles, four were finally included in the qualitative analysis (Fig. 1).

### Characteristics of the included studies

Table 1 describes in detail the four included studies [20–23], which targeted influenza virus infection. All studies were published between 2007 and 2020 and were from four different continents. Three studies used mathematical models [20–22] to simulate single- [20, 21] or multiple-direction flights [22].

**Table 1:** Characteristics of studies included in the qualitative synthesis

Author, year of publication	Type of study	Type of viral infection	Setting (no. of passengers)	Preventive measures		Outcome(s)	Main result(s)	Quality level (score)^a^
				Facemask use/hand hygiene	Other measures			
Caley, 2007 [20]	Mathematical model	Influenza	Flight (10–400)	Facemask use	Border screening, immediate presentation following symptom onset, flight-base quarantining	Time (days) of epidemic onset delay in an infection-free region following importation through air travel	Median time delay (days) increased from 57 to 79 days at an R_0_ of 1.5 and from 17 to 20 days at an R_0_ of 3.5	Not available
Gupta, 2012 [21]	Mathematical model	Influenza	Flight (21)	N95 respirator^b^ use	None	Infection probability	Infection probability was reduced from 15 to 0% (at 103 quanta per hour^c^) or from 100 to 55% (at 5226 quanta per hour)	Not available
Nicolaides, 2020 [22]	Mathematical model	Influenza	Flights moving through a network of (unspecified) international airports	Increasing the percentage of hand hygiene for air-traveling people at any time from 20% (i.e., one out of five people) to 30, 40, 50%, or 60%	None	Infection prevalence and total square displacement (TSD)	Infection prevalence and TSD were reduced by 18.2–55.4% and 23.7–69.1%, respectively	Not available
Zhang, 2013 [23]	Case-control study	Influenza A (H1N1)	Flight 1 (274) Flight 2 (144)	Facemask use and hand hygiene	None	Risk for infection transmission	Wearing a mask during flight reduced the infection transmission risk (OR, 0.0; 95% CI, 0.0–0.7) No difference in hand-hygiene compliance between infected and uninfected passengers	Medium (6)

^a^The quality of evidence for aircraft-related transmission was evaluated using the bias assessment tool proposed by Leitmeyer et al. [19]. Based on a score assignment, each study was categorized as having a low (0–3 points), medium (4–6 points), or high (7–9 points) level of evidence

^b^N95 is a respiratory protective device designed to achieve a very close facial fit and very efficient filtration of airborne particles (i.e., droplets containing an infectious agent)

^c^“Quantum” is a unit of measure that defines the amount of infectious material able to infect 1 − (1/e) (i.e., 63.2%) of the people in an enclosed space [21]

In one study, Caley et al. [20] simulated a 12-h flight per day with 10 to 400 passengers traveling from an epidemic region to a still infection-free region. In the second study, Gupta et al. [21] simulated a 4-h flight in which a fully occupied twin-aisle cabin carried the index passenger occupying the center seat of the cabin. The twenty passengers around the index passenger (six in the same row and seven in the front and back rows) represented the study population. In the third study, Nicolaides et al. [22] simulated flights moving through a large network of 120 international airports to mimic the global spread of viral disease.

Unlike the above three studies, the study by Zhang et al. [23] described the contact tracing of a real influenza outbreak that involved two flights during the 2009 influenza A H1N1 pandemic. One flight carried 274 passengers from New York City (United States) to Hong Kong (China), with a stopover in Vancouver. Sixty-three passengers, including the index patient, continued to travel on a connection flight from Hong Kong to Fuzhou (China), which carried 144 passengers in total. Contact tracing identified eight secondary influenza A H1N1 cases, seven in Fuzhou and one in Hong Kong, and all the infected passengers shared the flight from New York to Hong Kong, where transmission could have taken place. The basic reproductive number (R_0_) was provided in two studies and was 1.5–3.5 in one study [20] and 3.0 in another study [21].

### Studies evaluating the use of facemasks as a preventive measure

Three studies evaluated the use of facemasks to prevent viral transmission during air travel [20, 21, 23]. Gupta et al. [21] specifically analyzed N95 respirator use, whereas no details on the type of facemask were provided in the other two studies [20, 23].

Caley et al. [20] explored several variables that might affect the time delaying epidemic onset in an infection-free region following importation through air travel. The authors found that maximal compliance with facemask use and other nonpharmaceutical prevention measures (i.e., border screening, flight-based quarantining, or immediate presentation at the onset of symptoms) had less effect than the number of travelers per day, with a modest impact on R_0_ values. For example, during a 12-h travel of 400 passengers per day, facemask use increased the median time delay from 57 to 79 days at an R_0_ value of 1.5 and from 17 to 20 days at an R_0_ value of 3.5. Conversely, adopting facemask use together with another nonpharmaceutical prevention measure and reducing passenger numbers from 400 to 10 per day delayed the time both to 125 days at an R_0_ value of 1.5 and to 26 days at an R_0_ value of 3.5.

In the study by Gupta et al. [21], a computational fluid-dynamic simulation allowed estimation of the quantity and distribution of influenza virus particles of a single-cough exhalation (measured as “quanta” per hour). The effect of the N95 respirator on the risk of infection was evaluated as a function of the inhaled influenza virus particles. The infection probability wearing the N95 respirator was reduced from 15% (3/20) to 0% (0/20) at 103 exhaled quanta per hour or from 100% (20/20) to 55% (11/20) at 5226 exhaled quanta per hour.

In the study by Zhang et al. [23], a case-control analysis showed that wearing a facemask during flight was a significant protective factor against influenza A H1N1 infection on flight from New York City to Hong Kong. Consequently, none of the nine infected passengers compared to 15 (47%) of 32 healthy control passengers wore a facemask (odds ratio, 0.0; 95% confidence interval, 0–0.7).

### Studies evaluating hand hygiene as a preventive measure

Two studies evaluated hand hygiene as a strategy to prevent viral transmission during air travel [22, 23]. In the study by Nicolaides et al. [22] using Monte Carlo simulation, four hand-hygiene scenarios were hypothesized. In one scenario, increasing the percentage of people cleaning their hands at any time in all airports from 20% (i.e., one out of five people) to 30, 40, 50%, or 60%, allowed reduction of the infection prevalence by 18.2, 33, 45.2, and 55.4%, respectively. Similarly, the total square displacement of infected people—i.e., the measure of airports’ power to spread a disease across the globe—was reduced by 23.7, 43.4, 58.6, and 69.1%, respectively. The three other scenarios explored the effect of a less expensive and more contained strategy, i.e., hand-hygiene implementation only in a subset of busier airports, on the aforementioned outcomes, thus leading to similar results.

In contrast, the study by Zhang et al. [23] did not find significant differences in self-assessed hand-hygiene compliance (which consisted of hand washing after toilet use or hand cleaning by wet towel before eating) between case and control passengers. In both passenger groups, high rates of compliance were reported, namely, 100% (9/9 and 32/32, respectively) for washing hands after toilet use and 89% (8/9) and 91% (29/32) for cleaning hands before eating.

### Quality of included studies

The quality of evidence for aircraft-related transmission was assessed for only one study (i.e., that based on real outbreak data) [23]. We assigned positive points to the following criteria: index case classification (+ 1 point), secondary case definition (+ 2), contact tracing strategy (+ 2), and completeness of follow-up (+ 1). Zero points were assigned to the timeliness of contact tracing or the alternative exposure means for infection other than the flight, which were both considered criteria during investigation. Thus, the study was judged as having a medium level of evidence (Table 1).

## Discussion

Despite our extensive literature search on the topic, we were able to summarize the results of only four recently published studies that tried to investigate the potential of practices, such as facemask use or hand hygiene, in limiting the transmission of influenza virus-laden droplets and aerosols in aircraft. While the results from three studies [20–22] were probabilistic concerning the first practice, the case-control study by Zhang et al. [23] showed that not wearing a facemask was associated with an increased risk of infection in a confined space, such as the aircraft cabin during a flight. Less convincing are the results from these studies concerning the hand washing of passengers in airport/aircraft settings [22, 23]. Again, in the study by Zhang et al. [23], passengers in both the case and control groups appeared to be equally compliant in hand hygiene, with 100% of passengers claiming to have washed their hands after using the toilet, which may be a receptacle of virus-contaminated fomites [24]. Although self-inoculation of the nasal mucosa by contaminated hands is a well-documented mode of influenza virus transmission [25], aerosols or droplets represent the primary source of direct transmission from influenza (or other respiratory) virus-infected persons [24]. Therefore, we contextualized the findings from this systematic review to the current growing evidence on COVID-19 transmission/prevention concerns, which include the prospect of common practices (i.e., wearing facemasks) to control and prevent SARS-CoV-2 transmission in confined air travel-related spaces [10, 17].

As seen with influenza virus [26], to which SARS-CoV-2 has expressly been compared [27], surgical masks and N95 respirators became popular with severe respiratory syndromes due to severe acute respiratory syndrome coronavirus (SARS-CoV), Middle East respiratory syndrome coronavirus (MERS-CoV), and SARS-CoV-2, particularly in healthcare centers [28]. Leung et al. [29] detected viral RNA from three viruses (influenza, rhinovirus, and coronavirus) in respiratory droplets (30, 26, and 28%) and aerosols (40, 35, and 56%), respectively, which were collected from virus-infected participants while not wearing a surgical mask. In another study, Radonovich et al. [30] showed that N95 respirators were equally effective as surgical masks for preventing viral respiratory infections among healthcare personnel. Very recently, Jayaweera et al. [28] hypothesized the trajectories of droplets and aerosols from SARS-CoV-2-infected passengers seated in an aircraft who coughed with a surgical mask, with an N95 respirator, or without a facemask. When an infected passenger coughs, droplets and aerosols diffuse mainly forward—affecting passengers up to five rows ahead—but also backward—affecting passengers one row behind—and sideways—affecting the passenger seated next to the infected person. Consequently, in the absence of social distancing, wearing a surgical mask/N95 respirator allows filtration of 20 to 30% of the SARS-CoV-2 load eventually present in the cabin air [28]. Similarly, based on data about the quanta generation rate and facemask efficiency, Wang et al. [31] estimated the inflight SARS-CoV-2 infection probability (assuming aerosol transmission) for a range of possible scenarios (e.g., severe [100 quanta/h] or mild [5 quanta/h]) within the economy class and business class sections of the aircraft. For a 12-h flight, the average infection probability in the economy class section varies from 0.8% (mild scenario) to 10.8% (severe scenario) without facemasks and decreases by approximately 73%/32% with high/low efficiency masks [31].

Consistent with current observations [10, 16, 17], we suggest a strategy aimed at controlling/preventing the spread of airborne viral diseases, including SARS-CoV-2, through air travel. This strategy will necessarily include hygiene measures (i.e., wearing facemasks or promoting personal hygiene), even though other measures (i.e., implementing effective preflight screening or promoting distancing while boarding and deplaning) may be adopted before and during boarding to prevent virus transmission. All these measures are underscored in international guidelines from the European Centre for Disease Prevention and Control (ECDC) or the WHO agencies. Below, we provide a summary of key measures that may mitigate the travel-related spread of COVID-19.

Modern aircraft are equipped with high-efficiency particulate air (HEPA) filters that remove almost all particles of bacteria, fungi, and viruses, which range from 0.1 μm to 0.3 μm in diameter, from circulating air in aircraft cabins [17]. Although the SARS-CoV-2 particle diameter is smaller, virus-laden droplets and aerosols are larger than the 5–10 μm size required for capture by HEPA filters [17]. Therefore, viral transmission in the aircraft cabin may occur due to person-to-person contact, implying that passengers should be reminded about facemask wearing, hand hygiene (including avoiding touching seats and other cabin surfaces), and reducing movement in the cabin [16, 17, 32]. Consequently, aircraft should be sanitized prior to boarding, and adequate ventilation should be ensured at all times. Social distancing, whenever possible, is also recommended, as well as simplified onboard services and minimized carry-on luggage [16, 17, 32]. Another issue connected with the use of facemasks is limiting their removal (possibly to under 15 min) during eating to minimize the potential risk of virus exposure [17]. This risk might be null in short-haul flights if airlines consider limiting or avoiding meal and drink services on these flights [17].

Transmission of infectious diseases among air travelers can occur in locations other than in commercial aircraft, e.g., from the entry in the first airport to the departure from the last [32]. Therefore, the International Civil Aviation Organization (ICAO) guidance for safe air travel in the context of COVID-19 embraces multiple layers of protection that involve airports as well as aircraft [16]. Importantly, individuals (including the crew) with viral respiratory infection—fever and/or respiratory symptoms (i.e., cough or sneeze)—should not travel until full remission [32]. In the case of SARS-CoV-2 infection, at least two consecutively negative nasopharyngeal swabs from the index passenger are required to protect other passengers from contracting the disease. During an ongoing pandemic, all passengers, including the crew, regardless of their country of departure, should be screened. Instead, during an epidemic, it is enough to screen passengers departing from WHO-identified viral infection areas [33]. Screening includes, in addition to temperature control—which is thought to have limited effect as a screening method alone—observation of respiratory symptoms [10]. It is recommended that passengers use hand disinfection gel and surgical masks, which should be made available during the boarding procedure. Surgical masks should be mandatory during flight, as possibly infected passengers may be asymptomatic (and then develop a fever) or may use antipyretics to suppress fever [16].

It is also recommended that flight attendants observe passengers for respiratory symptoms at all times [33]. On medium- and long-haul flights, temperature control may be repeated. Hand disinfection should be ensured before distributing foods and should be repeated as well. Soap and disinfectant should be available at toilets at all times. Passengers presenting with fever and/or respiratory symptoms during a flight should be socially distanced from other passengers, moving other passengers several seats away from the infected passenger [32]. In the case of a full flight, N95 respirators should be handed to the infected passenger, including those around him/her. Flight attendants caring for infected persons should wear gloves and a facemask [32].

To increase correct behavior and compliance, easy-to-follow health information should be provided on board, e.g., video clips about hand hygiene and facemask use and disposal [34]. Questionnaires at arrival, e.g., health declarations with contact details of passengers, are recommended to permit contact tracing and risk assessment [33] and, importantly, provide insights into how hand hygiene and facemask use mitigate viral infection risk. It is important to recall that the effect of facemasks is curtailed when they are not appropriately used [17] and that distributing free masks promotes facemask use [17].

One intrinsic limitation of the present review is the very small number of included studies. Consequently, the main findings referred to N95 respirators, which are advised for healthcare workers, and not to surgical masks, which are advised to be worn in public spaces, such as those frequented by air travelers. Likewise, the main findings referred to mathematical model settings, which limits their applicability to real-time settings. Additionally, the self-reporting of compliance with hand hygiene in one of the included studies somewhat limits the strength of conclusions drawn from the present review. Immediately after the time of our writing (i.e., early December 2020), published literature on the risk for inflight transmission of COVID-19 has become populated with studies (one of which was cited here [31]). Therefore, it is possible that we have unintentionally omitted relevant information on a public health topic continuously prompted by the escalation of COVID-19 cases worldwide.

## Conclusions

Adoption of facemask use combined with other hygiene measures may help to minimize the chance of SARS-CoV-2 spread by air travelers. This proposal is in line with the current recommendations by IATA that pose facemask wearing onboard as the most visible component in a multilayered approach to prevent inflight transmission of COVID-19 [16]. Not surprisingly, in March 2020, the WHO recommended using facemasks to manage onboard persons with respiratory symptoms compatible with COVID-19 [32]. In particular, hand hygiene should be emphasized to slow the spread of respiratory diseases, including COVID-19 [35]. However, more efforts are required before hygiene measures become an integral part of standard procedures in aircraft. These include extensive resources and preparation for implementing and/or increasing passenger compliance with such measures. Moreover, understanding the effectiveness of different measures aimed at controlling/preventing infectious diseases, such as the ongoing COVID-19 pandemic, requires rigorous and well-designed studies in the future.

## Supplementary Information

**Additional file 1.**

## Data Availability

All data generated or analyzed during this study are included in this published article and its additional file 1.

## Abbreviations

HICPAC: Healthcare infection control practices advisory committee
WHO: World health Organization
SARS-CoV-2: Severe acute respiratory syndrome coronavirus 2
COVID-19: Coronavirus disease 2019
SARS: Severe acute respiratory syndrome
IATA: International air transport association
PRISMA: Preferred reporting items for systematic reviews and meta-analyses
PICOS: Participants, intervention, comparator, outcome, and study design
SARS-CoV: Severe acute respiratory syndrome coronavirus
MERS-CoV: Middle East respiratory syndrome coronavirus
*ECDC*: European centre for disease prevention and control
HEPA: High-efficiency particulate air
ICAO: International civil aviation Organization

## References

[1] Gralton J, Tovey E, McLaws ML, Rawlinson WD (2011). The role of particle size in aerosolised pathogen transmission: a review. J Inf Secur.

[2] Boone SA, Gerba CP (2007). Significance of fomites in the spread of respiratory and enteric viral disease. Appl Environ Microbiol.

[3] Brankston G, Gitterman L, Hirji Z, Lemieux C, Gardam M (2007). Transmission of influenza a in human beings. Lancet Infect Dis.

[4] Siegel JD, Rhinehart E, Jackson M, Chiarello L (2007). Health care infection control practices advisory committee. 2007 guideline for isolation precautions: preventing transmission of infectious agents in health care settings. Am J Infect Control.

[5] Infection prevention and control of epidemic- and pandemic-prone acute respiratory infections in health care. Geneva: World Health Organization; 2014. https://www.ncbi.nlm.nih.gov/books/NBK214359. Accessed 6 December 2020. 24983124

[6] Gallaher WR (2009). Towards a sane and rational approach to management of influenza H1N1 2009. Virol J.

[7] Coronaviridae Study Group of the International Committee on Taxonomy of Viruses (2020). The species severe acute respiratory syndrome-related coronavirus: classifying 2019-nCoV and naming it SARS-CoV-2. Nat Microbiol.

[8] Non-pharmaceutical public health measures for mitigating the risk and impact of epidemic and pandemic influenza. Geneva: World Health Organization; 2019. https://www.who.int/influenza/publications/public_health_measures/publication/en. Accessed 6 December 2020.

[9] Qualls N, Levitt A, Kanade N, Wright-Jegede N, Dopson S, Biggerstaff M (2017). Community mitigation guidelines to prevent pandemic influenza – United States, 2017. MMWR Recomm Rep.

[10] Guidelines for non-pharmaceutical interventions to reduce the impact of COVID-19 in the EU/EEA and the UK. Stockholm: European Centre for Disease Prevention and Control; 2020. https://www.ecdc.europa.eu/sites/default/files/documents/covid-19-guidelines-non-pharmaceutical-interventions-september-2020.pdf. Accessed 6 December 2020.

[11] Elder AG, O'Donnell B, McCruden EA, Symington IS, Carman WF (1996). Incidence and recall of influenza in a cohort of Glasgow healthcare workers during the 1993-4 epidemic: results of serum testing and questionnaire. BMJ.

[12] Miller JM, Tam TW, Maloney S, Fukuda K, Cox N, Hockin J (2000). Cruise ships: high-risk passengers and the global spread of new influenza viruses. Clin Infect Dis.

[13] Troko J, Myles P, Gibson J, Hashim A, Enstone J, Kingdon S, Packham C, Amin S, Hayward A, van-Tam JN (2011). Is public transport a risk factor for acute respiratory infection?. BMC Infect Dis.

[14] Moser MR, Bender TR, Margolis HS, Noble GR, Kendal AP, Ritter DG (1979). An outbreak of influenza aboard a commercial airliner. Am J Epidemiol.

[15] Olsen SJ, Chang HL, Cheung TY, Tang AF, Fisk TL, Ooi SP (2003). Transmission of the severe acute respiratory syndrome on aircraft. N Engl J Med.

[16] Research points to low risk for COVID-19 transmission inflight. International Air Transport Association; 2020. https://www.iata.org/en/pressroom/pr/2020-10-08-02. Accessed 18 March 2021.

[17] Khatib AN, Carvalho AM, Primavesi R, To K, Poirier V (2020). Navigating the risks of flying during COVID-19: a review for safe air travel. J Travel Med.

[18] Liberati A, Altman DG, Tetzlaff J, Mulrow C, Gøtzsche PC, Ioannidis JP (2009). The PRISMA statement for reporting systematic reviews and meta-analyses of studies that evaluate health care interventions: explanation and elaboration. PLoS Med.

[19] Leitmeyer K, Adlhoch C (2016). Review article: influenza transmission on aircraft: a systematic literature review. Epidemiology..

[20] Caley P, Becker NG, Philp DJ (2007). The waiting time for inter-country spread of pandemic influenza. PLoS One.

[21] Gupta JK, Lin CH, Chen Q (2012). Risk assessment of airborne infectious diseases in aircraft cabins. Indoor Air.

[22] Nicolaides C, Avraam D, Cueto-Felgueroso L, González MC, Juanes R (2020). Hand-hygiene mitigation strategies against global disease spreading through the air transportation network. Risk Anal.

[23] Zhang L, Peng Z, Ou J, Zeng G, Fontaine RE, Liu M, Cui F, Hong R, Zhou H, Huai Y, Chuang SK, Leung YH, Feng Y, Luo Y, Shen T, Zhu BP, Widdowson MA, Yu H (2013). Protection by face masks against influenza a(H1N1)pdm09 virus on trans-Pacific passenger aircraft, 2009. Emerg Infect Dis.

[24] Morawska L (2006). Droplet fate in indoor environments, or can we prevent the spread of infection?. Indoor Air.

[25] Tellier R (2009). Aerosol transmission of influenza A virus: a review of new studies. J R Soc Interface.

[26] Long Y, Hu T, Liu L, Chen R, Guo Q, Yang L, Cheng Y, Huang J, du L (2020). Effectiveness of N95 respirators versus surgical masks against influenza: a systematic review and meta-analysis. J Evid Based Med.

[27] Petersen E, Koopmans M, Go U, Hamer DH, Petrosillo N, Castelli F, et al. Comparing SARS-CoV-2 with SARS-CoV and influenza pandemics. Lancet Infect Dis. 2020;(20):S1473, 30484–3099, 30489. 10.1016/S1473-3099(20)30484-9. 10.1016/S1473-3099(20)30484-9 PMC7333991 32628905

[28] Jayaweera M, Perera H, Gunawardana B, Manatunge J (2020). Transmission of COVID-19 virus by droplets and aerosols: a critical review on the unresolved dichotomy. Environ Res.

[29] Leung NHL, Chu DKW, Shiu EYC, Chan KH, McDevitt JJ, Hau BJP (2020). Respiratory virus shedding in exhaled breath and efficacy of face masks. Nat Med.

[30] Radonovich LJ, Simberkoff MS, Bessesen MT, Brown AC, Cummings DAT, Gaydos CA, Los JG, Krosche AE, Gibert CL, Gorse GJ, Nyquist AC, Reich NG, Rodriguez-Barradas MC, Price CS, Perl TM, for the ResPECT investigators (2019). N95 respirators vs medical masks for preventing influenza among health care personnel: a randomized clinical trial. JAMA.

[31] Wang Z, Galea ER, Grandison A, Ewer J, Jia F. Inflight transmission of COVID-19 based on experimental aerosol dispersion data. J Travel Med. 2021:taab023. 10.1093/jtm/taab023. 10.1093/jtm/taab023 PMC7928737 33615383

[32] Operational considerations for managing COVID-19 cases or outbreak in aviation. Interim guidance 18 March 2020. Geneva: World Health Organization; 2020. https://apps.who.int/iris/bitstream/handle/10665/331488/WHO-2019-nCoV-Aviation-2020.1-eng.pdf?sequence=1&isAllowed=y. Accessed 18 March 2021.

[33] Considerations for implementing a risk-based approach to international travel in the context of COVID-19. Interim guidance 16 December 2020. Geneva: World Health Organization; 2020. https://www.who.int/publications/i/item/WHO-2019-nCoV-Risk-based-international-travel-2020.1. Accessed 18 March 2021.

[34] Advice on the use of masks in the context of COVID-19. Interim guidance 5 June 2020. Geneva: World Health Organization; 2020. https://www.who.int/publications/i/item/advice-on-the-use-of-masks-in-the-community-during-home-care-and-in-healthcare-settings-in-the-context-of-the-novel-coronavirus-(2019-ncov)-outbreak. .

[35] Jefferson T, Del Mar CB, Dooley L, Ferroni E, Al-Ansary LA, Bawazeer GA, et al. Physical interventions to interrupt or reduce the spread of respiratory viruses. Cochrane Database Syst Rev. 2020;(11). 10.1002/14651858.CD006207.pub5. 10.1002/14651858.CD006207.pub5 PMC8094623 33215698

