# Supplementary material for: Hand hygiene and facemask use to prevent droplet-transmitted viral diseases during air travel: a systematic literature review
Source: BMC Public Health. 2021 Apr 20;21:760. doi: 10.1186/s12889-021-10814-9 (PMC8056366; doi:10.1186/s12889-021-10814-9)
Supplement: Supplementary file 1 — Additional file 1. [file 12889_2021_10814_MOESM1_ESM.doc]

Literature Search strategies

**PubMed**

#1 (((((("aerospace medicine"[MeSH Terms] OR “aircraft”[MeSH Terms] OR “aviation”[MeSH Terms] OR “airports”[MeSH Terms] OR aircraft*[tiab] OR aeroplane*[tiab] OR airline*[tiab] OR flight*[tiab] OR aircrew[tiab] OR airflight*[tiab] OR airplane*[tiab] OR aviation[tiab] OR airport*[tiab] OR aeroport*[tiab] OR "aero transport"[tiab] OR "airport"[tiab] OR stewart[tiab] OR stewardess[tiab] OR inflight[tiab] OR "in-flight"[tiab] OR "cabin crew"[tiab] OR cabin[tiab] OR cabins[tiab] OR ((travel*[tiab] OR “travel”[MeSH Terms] OR transport*[tiab] OR journey*[tiab] OR trip[tiab] OR trips[tiab]) and air[tiab]) OR ((plane[tiab] OR planes[tiab]) AND (air[tiab] OR travel*[tiab] OR “travel”[MeSH Terms] OR transport*[tiab] OR journey*[tiab] OR trip[tiab] OR trips[tiab])) OR ((passenger*[tiab] OR crew[tiab] OR traveller*[tiab] OR personnel[tiab] OR staff[tiab]) and (flying[tiab] OR fly[tiab] OR air[tiab])))))

#2 ("communicable diseases"[MeSH Terms] OR “transmission” [tiab] OR “infection”[tiab] OR “fever”[tiab] OR airborne OR droplet*)

#3 ("time of flight"[tiab] AND spectrometry [tiab])

#4 #1 AND #2

#5 #4 NOT #3

#6 “Personal protective equipment” [MeSH Terms] OR mask*[tiab]

#7 “Hand hygiene” [MeSH Terms] OR “hand washing” [tiab] OR “handwashing” [tiab] OR “hand disinfection” [tiab] OR “hydroalcoholic solution” [tiab] OR “infection control” [MeSH Terms] OR “preventive measures” [MeSH Terms]

#8 #6 OR #7

#9 #5 AND #8

**Web of Science**

1# Ts=("aerospace medicine" or "aircraft" or "aviation" or "airports" or aircraft* or aeroplane* or airline* or flight* or aircrew or airflight* or airplane* or aviation or airport* or aeroport* or "aero transport" or "airport" or stewart or stewardess or inflight or "in-flight" or "cabin crew" or cabin or cabins or ((travel* or "travel" or transport* or journey* or trip or trips) and air) or ((plane or planes) and (air or travel* or "travel" or transport* or journey* or trip or trips)) or ((passenger* or crew or traveller* or personnel or staff) and (flying or fly or air)))

2# TS=((Communicable disease) OR “transmission” OR “infection” OR “fever” OR airborne OR droplet*)

3# TS=(“Personal protective equipment” OR mask* OR “Hand hygiene” OR “hand washing” OR “handwashing” OR “hand disinfection” OR “hydroalcoholic solution”) OR “infection control” [MeSH Terms] OR “preventive measures” [MeSH Terms]

1# AND 2# AND 3#

**Scopus**

TITLE-ABS-KEY("aerospace medicine" or "aircraft" or "aviation" or "airports" or aircraft* or aeroplane* or airline* or flight* or aircrew or airflight* or airplane* or aviation or airport* or aeroport* or "aero transport" or "airport" or stewart or stewardess or inflight or "in-flight" or "cabin crew" or cabin or cabins or ((travel* or "travel" or transport* or journey* or trip or trips) and air) or ((plane or planes) and (air or travel* or "travel" or transport* or journey* or trip or trips)) or ((passenger* or crew or traveller* or personnel or staff) and (flying or fly or air))) AND TITLE-ABS-KEY((Communicable disease) OR “transmission” OR “infection” OR “fever” OR airborne OR droplet*) AND TITLE-ABS-KEY (“Personal protective equipment” OR mask* OR “Hand hygiene” OR “hand washing” OR “handwashing” OR “hand disinfection” OR “hydroalcoholic solution”)

**Gray Literature**

- Web search was conducted by using Google advanced search (limiting results to first twenty pages) with the following keywords: ~airplane, ~flight, ~transmission, ~infection, “hand hygiene”, “mask”, “personal protective equipment”.

- Targeted web search were conducted by using Google advanced search and by hand-search at the following sites: www.who.int, https://www.cdc.gov/, https://www.ecdc.europa.eu/en, https://www.nhs.uk/, https://www.escmid.org, https://www.health.gov.au, https://www.gov.uk/government/organisations/public-health-england, https://www.canada.ca/en/public-health.html.

- Study protocols and trials were searched on the following sites: US ClinicalTrials.gov, WHO International clinical trials registry platform search portal, Metaregister of controlled trials. Dissertations, Theses, and Academic Papers were searched on Opengrey. The following keywords were used: infection, communicable disease, aircraft, airplane, travel, hand hygiene, mask.
